# Attenuated PER3 amplitude in hair follicles is associated with sleep irregularity in schizophrenia

**DOI:** 10.1038/s41398-026-04295-w

**Published:** 2026-07-29

**Authors:** Mikkel E. I. Kolind, Rikke Kruse, Jan C. Brønd, Afsaneh M. Nejad, Lasse K. Bak, Charlotte S. Larsen, Kurt Højlund, Christoph P. Beier, Elsebeth Stenager, Claus B. Juhl

**Affiliations:** 1 Steno Diabetes Center Odense, Odense University Hospital, Odense, Denmark; 2 Department of Regional Health Research, University of Southern Denmark, Odense, Denmark; 3 Open Patient data Explorative Network - OPEN, University of Southern Denmark, Odense, Denmark; 4 Department of Endocrinology, University Hospital of Southern Denmark, Esbjerg, Denmark; 5 Department of Clinical Research, University of Southern Denmark, Odense, Denmark; 6 Department of Sports Science and Clinical Biomechanics, University of Southern Denmark, Odense, Denmark; 7 Department of Mathematics and Computer Science, University of Southern Denmark, Odense, Denmark; 8 Department of Clinical Biochemistry, Copenhagen University Hospital, Rigshospitalet, Glostrup, Denmark; 9 Translational Research Center (TRACE), Copenhagen University Hospital – Rigshospitalet, Glostrup, Denmark; 10 Department of Drug Design and Pharmacology, University of Copenhagen, Copenhagen, Denmark; 11 Department of Neurology, Odense University Hospital, Odense, Denmark; 12 Research Unit, Mental Health South West, Department of Regional Health Research, University of Southern Denmark, Aabenraa, Denmark

**Keywords:** Schizophrenia, Physiology, Predictive markers

## Abstract

Circadian dysregulation is prevalent in both schizophrenia and obesity, yet the molecular mechanisms underpinning these disturbances remain unclear. We examined circadian variation in clock gene expression using repeated hair follicle self-sampling across two days in 66 adults: 22 with schizophrenia and obesity (SCH-OB), 22 with obesity but no psychiatric disorder (OB), and 22 normal-weight controls (LEAN). Gene expression was quantified by qPCR, and complementary markers included salivary cortisol and melatonin, oral pH and temperature, and accelerometer-derived sleep metrics. Evidence of rhythmic expression was detected in five of six clock genes. *PER3* amplitude was significantly reduced in SCH-OB compared with both OB and LEAN (*p* < 0.01) and correlated positively with sleep regularity (*p* = 0.006). Cortisol rhythms were preserved across groups, whereas melatonin amplitude was attenuated in SCH-OB relative to LEAN (*p* < 0.05). Hair follicle sampling proved highly feasible, with >98% compliance. These findings indicate that individuals with schizophrenia exhibit dampened circadian rhythms in *PER3* expression that parallel irregular sleep patterns, supporting hair follicle–based sampling as a practical tool for assessing molecular rhythm parameters in real-world conditions.

## Introduction

Circadian rhythms regulate physiological processes such as sleep–wake cycles, hormone release, and energy metabolism, as well as psychological functions including mood stability and decision-making. Disruptions to this finely tuned system are associated with negative health consequences, including metabolic disorders, obesity, and psychiatric illnesses [1–5].

Mental disorders, like schizophrenia, are associated with alterations in circadian rhythms and sleep–wake patterns [6, 7], which contribute to a range of negative psychological outcomes, including increased anxiety, depressive symptoms, emotional dysregulation, impaired cognitive function, and nighttime rumination, making individuals more vulnerable to persistent negative mood states [4, 8, 9]. These effects may form a self-perpetuating cycle, where poor sleep exacerbates psychological symptoms, which may in turn further disrupt sleep continuity and quality [2]. Additionally, obesity is associated with both mental disorders and circadian dysregulation [1, 3]. Together, these interconnections form a complex interplay where each factor—mental disorders, circadian dysregulation, and obesity—can exacerbate the others, increasing the risk of metabolic and psychiatric comorbidities.

At the molecular level, circadian rhythms are regulated by a core network of clock genes. These include the Period (*PER1*, *PER2*, *PER3*), Nuclear Receptor Subfamily 1 Group D (*NR1D1*, *NR1D2*), and Brain and Muscle ARNT-Like 1 (*BMAL1*) genes, which interact with other clock genes through transcriptional–translational feedback loops to generate self-sustaining rhythmic patterns of gene expression aligned with environmental cues [5]. Aberrant expression of these genes in peripheral blood cells has been linked to sleep disorders such as obstructive sleep apnea (OSA) and insomnia [10], as well as metabolic diseases like type 2 diabetes (T2D) [11], while altered expression in skeletal muscle has been associated with obesity [12]. Similarly, dysregulated expression of circadian genes in peripheral blood cells has been reported in depression [13] and schizophrenia [14].

As circadian rhythms are influenced by habitual behavior and routines [15], assessing circadian function in a home environment, where individuals maintain their typical schedules, may better capture how real-world behaviors and zeitgebers shape circadian rhythms in everyday settings, complementing controlled laboratory protocols designed to isolate endogenous rhythms. Previous studies investigating clock gene expression in the context of mental disorders have relied on animal models [16, 17], single time-point assessments [18] or data obtained under controlled laboratory conditions [13, 14]. This is the first study to assess clock gene expression through repeated sampling in a naturalistic, home environment in individuals with obesity and mental disorders.

Hair follicle sampling for the analysis of clock gene expression was introduced by Akashi et al. [19]. Unlike blood or fibroblast-based methods that require controlled laboratory conditions, hair follicle sampling offers a minimally invasive, repeatable, and ecologically valid approach that is compatible with study designs prioritizing real-world conditions. This makes it particularly well-suited for studies investigating the interplay between mental disorders, circadian dysregulation, and real-world behavioral patterns [20].

In the present study, we aimed to investigate whether individuals with schizophrenia exhibit distinct patterns of clock gene expression compared with lean controls and weight-matched obese controls. Circadian rhythms were examined using hair follicle–derived gene expression (qPCR), salivary cortisol and melatonin, and accelerometer-derived sleep patterns under ecologically valid home conditions. We quantified sampling adherence and compared self-sampling with assisted sampling by study personnel. The matched case–control design included three groups: SCH-OB (BMI ≥ 30 kg/m²), OB (BMI ≥ 30 kg/m²; no mental illness), and LEAN (BMI < 25 kg/m²; no psychiatric or sleep disorders).

## Material and methods

This was an observational study with repeated measures across two consecutive days. Data collection included hair follicle sampling, saliva sampling, wrist-worn accelerometry, oral pH and temperature, and standardized questionnaires. Procedures comprised both a baseline clinical visit (anthropometry, questionnaires, device handout) and home-based self-sampling. Full procedural details are described in our published protocol [21].

### Study participants

Sixty-six participants were enrolled and assigned to one of three groups: (1) individuals with schizophrenia and obesity (SCH-OB), (2) individuals with obesity but no psychiatric diagnosis and no psychotropic medication (OB), and (3) normal weight individuals without psychiatric diagnosis and without psychotropic medication use (LEAN). The OB group served as an obesity-only comparator, allowing obesity-associated differences (OB vs LEAN) to be distinguished from schizophrenia-with-obesity differences (SCH-OB vs OB and SCH-OB vs LEAN). All groups were matched based on age (±5 years) and sex. SCH-OB and OB were additionally group-wise matched on BMI (±3 kg/m²) and OSA status. The study was preregistered on ClinicalTrials.gov (Identifier: NCT05413486), which originally included a fourth, unmatched comparison group of individuals with bipolar disorder and obesity. However, due to recruitment constraints, the final study design focused on the three matched groups described above.

### Gene expression analysis

Procedures for hair follicle sampling, RNA extraction, and cDNA synthesis were conducted as described in our published protocol [21]. Briefly, total RNA was extracted from plucked beard or scalp hair follicles collected at four time points (0, 6, 12 h after waking, and just before bed) across two consecutive days using a RNeasy Micro kit (QIAGEN, Hilden, Germany) according to the manufacturer’s instructions. The RNA concentration was quantified using a NanoDrop spectrophotometer (Thermo Fisher Scientific, Waltham, MA) after which RNA was reverse transcribed to cDNA using a high-capacity cDNA reverse transcription kit (Applied Biosystems, Foster City, CA) according to the manufacturer’s protocol. The qPCR was performed on a QuantStudio 5 real-time PCR system (Applied Biosystems, Foster City, CA) using pre-designed, commercially available TaqMan Custom Arrays against six core circadian clock genes *PER2* (Hs01007553_m1), *PER3* (Hs00213466_m1), *NR1D1* (Hs00253876_m1), *NR1D2* (Hs00233309_m1), *BMAL1* (Hs00154147_m1), and *RORA* (Hs00536545_m1) (all from Applied Biosystems, Foster City, CA). Gene expression was normalized to the geometric mean of two reference genes, *RPLP0* and *TBP*, selected based on expression stability across time points and study groups. All samples were run in triplicates, and the relative expression was calculated using the 2^–ΔCt method [22].

### Saliva analyses

Saliva samples were handled as described, and in accordance with our established procedure for home-sampling [21, 23]. Briefly, samples were collected at waking, 6 and 12 h after waking, every 2 h thereafter, 1 h before bed, and just before bed across two consecutive days. Immediately after collection, samples were refrigerated by participants and subsequently centrifuged within 72 h of sampling time. They were then stored at –80 °C until analysis. Cortisol levels were quantified using a chemiluminescence immunoassay (IDS-iSYS, Immunodiagnostic Systems, Tyne & Wear, United Kingdom; IS-4900), while melatonin concentrations were measured via ELISA (IBL International, Hamburg, Germany; RE54041). Internal quality control measures were in place and the inter-assay coefficient of variation (CV) for the melatonin assay was calculated at two levels to be 24% (2.3 ng/L) and 28% (11.8 ng/L) with an average intra-assay CV of 18% (at the 2.3 ng/L level). For the cortisol assay, the intra-assay CV was maximum 15% at three different levels (4.7, 12, and 48 nmol/L). When sample volume was insufficient for both hormonal analyses, cortisol measurement was prioritized. Salivary pH was assessed using a standard pH electrode. Additional statistics and information regarding cortisol and melatonin testing are available in Supplementary Figs. S7-9.

### Questionnaire data and anthropometry

Sleep quality was assessed using the Pittsburgh Sleep Quality Index (PSQI) [24], and chronotype the Morningness–Eveningness Questionnaire (MEQ) [25]. Anthropometric measurements were collected at the first clinical visit as previously described [21].

### Accelerometer derived sleep outcomes

Sleep variables were derived from wrist-worn accelerometer raw-data using R [26] and the open-source R package GGIR [27], developed by Van Hees et al. [28, 29]. For this study, accelerometry was used to calculate the Sleep Regularity Index (SRI), sleep duration, sleep onset, and sleep offset [30]. All data were processed using default GGIR parameters and summary variables were averaged across available valid days.

### Blinding

All laboratory tests and accelerometer-based assessments were conducted by personnel blinded to group membership.

### Statistics

To determine whether physiological variables and gene expression exhibited 24-hour rhythmicity, we applied linear mixed-effects cosinor models. For each outcome, we fitted two models: a nested model including only a random effect for subject (ID), and a full cosinor model with fixed-effect sine and cosine terms and a random intercept for subject ID. Likelihood ratio tests (LRTs) were used to compare the models. A significant LRT p-value indicated that the inclusion of rhythmic components improved model fit, consistent with the presence of a 24-hour sinusoidal rhythm and supporting the appropriateness of cosinor-based regression [31]. Changes in Akaike’s Information Criterion (ΔAIC = AIC_(null model)_- AIC_(full model)_) were used as a secondary indicator to quantify improvements in model fit [32]. To test for group differences in clock gene amplitude (SCH-OB, OB, LEAN), we applied mixed-effects cosinor models using the cosinor.lm function from the cosinoRmixedeffects R package [33]. These models included sine and cosine terms as fixed effects, subject ID as random effects and group as fixed effect. Post-hoc contrasts were used to estimate differences in amplitude, MESOR (midline estimating statistic of rhythm) and time of peak expression (acrophase) between groups. Pairwise comparisons were Tukey-adjusted to control the family-wise error rate within each outcome/parameter. Significance was assessed at α = 0.05. 95% CIs for model-derived parameters were obtained via parametric bootstrapping (n = 1000). At the individual level, we applied standard cosinor linear regression models with sine and cosine terms, and used F-tests to assess whether the rhythmic terms jointly contributed to model fit [31]. To examine whether sleep metrics or psychotropic medication dose (expressed as chlorpromazine equivalents (CPZeq) [34]) were associated with modulation of clock gene amplitude, we constructed a separate set of models in which the cosinor framework was extended to include interaction terms between PSQI, SRI, CPZeq and the harmonic predictors (sine and cosine terms). These models tested whether PSQI, SRI, or CPZeq modulated the amplitude of clock gene expression. For each gene, model-based estimates of amplitude were computed across the observed range of PSQI or SRI values. 95% CI’s were obtained via parametric bootstrapping (n = 1000), and p-values for the slope of the amplitude~predictor association were calculated from the bootstrap distribution and corrected for multiple comparisons [35]. Marginal and conditional R² values were computed using the MuMIn R package to summarize the proportion of variance explained by fixed effects alone and by the full model (including random effects), respectively. To deal with missing data, mixed-effects models incorporate all observed data, enhancing the robustness of the estimates [33]. All statistics were conducted in R version 4.3.1.

## Results

### High compliance and adequate RNA yield support feasibility of hair follicle sampling in schizophrenia

**Fig. 1:**
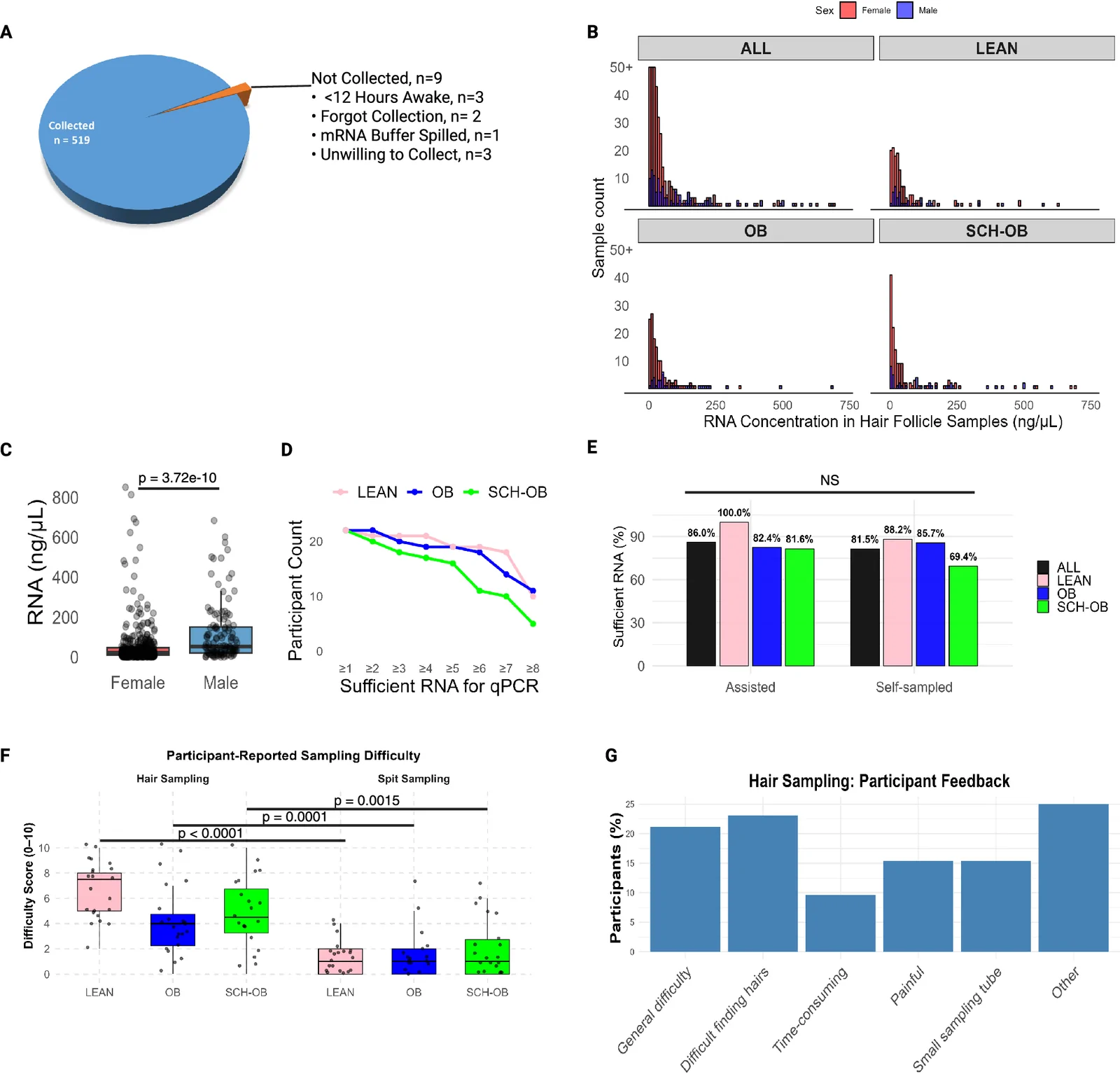
Hair follicle sampling compliance, RNA yield, and participant experience. (**A**) Sampling compliance. (**B**) Distribution of RNA concentrations (ng/µL) in hair follicle samples, shown by sex and by group. (**C**) RNA concentrations in individual hair follicle samples, stratified by sex; group differences were assessed using nonparametric Mann–Whitney U tests. (**D**) Number of participants with one to eight hair follicle samples containing sufficient RNA ( ≥ 8 ng/µL) for qPCR analysis, shown by group. (**E**) Proportion of samples with sufficient RNA, grouped by sampling method and participant group; group comparisons were assessed using Kruskal–Wallis tests. Assisted samples were carried out during home visits. (**F**) Participant-reported difficulty of hair follicle sampling, plotted alongside corresponding saliva sampling to allow comparison. Ratings range from 0 (not difficult) to 10 (very difficult). (**G**) Open-ended feedback rating. Responses were collected as free-text comments and subsequently categorized based on themes related to perceived difficulty with hair sampling. The “Other” category includes general remarks not directly related to sampling difficulty.

Participant compliance was high, with 519 out of the 528 initially planned samples collected by participants (Fig. 1). All samples were tested for RNA content. We found that a minimum RNA concentration of 8 ng/µL was required for qPCR analysis. The median RNA yield from samples was 26 (10; 62.8) ng/µL. Median yields were 28.5 (14; 62.2) ng/µL in LEAN, 27.0 (11.5; 54.2) ng/µL in OB, and 19.0 (6.0; 88.0) ng/µL in SCH-OB (Fig. 1B). While RNA yield did not significantly differ between groups, males had a higher median yield than females (Fig. 1C), and all participants had at least one sample with sufficient RNA for analysis (Fig. 1D). Participants were offered assisted sampling during home visits, and 93 samples were collected this way (38 in SCH-OB, 34 in OB, and 21 in the LEAN group). The proportion of samples with sufficient RNA was 86.0% and 81.5% when self-sampled and assisted by study personnel, respectively, and there was no significant difference between the two methods in RNA yield (Fig. 1E). After the conclusion of the two-day study period, the participants were asked to rate their experience of hair follicle sampling on a 0–10 rating scale with 0 indicating “not difficult” and 10 being “very difficult.” Saliva sampling was rated concurrently, and participants rated saliva sampling as significantly less difficult (Fig. 1F). In addition to the numerical ratings, the participants were given the opportunity to provide open-ended feedback. Responses were categorized in themes *post hoc*, with the most frequent themes including hair-plucking-related discomfort and difficulties locating suitable hairs (Fig. 1G).

### Participants

All participants were successfully matched on age and sex. Matching on OSA status was largely successful, though one participant’s OSA diagnosis changed post-enrollment. Three individuals were of non-Scandinavian descent: two in the OB group (one of Latin American and one of Eastern European origin) and one in the SCH-OB group (Eastern European origin). SCH-OB and OB groups were similar on non-matched anthropometric measures. Chronotype, as assessed by questionnaire, was comparable across groups, with approximately 80% of participants in each group classified as either moderate morning or intermediate types (Table 1). No participants withdrew or were lost to follow-up; all completed the study protocol.

**Table 1:** Participant Characteristics.

	SCH-OB (n = 22)	OB (n = 22)	LEAN (n = 22)
Female (n (%))	17 (77.3)	17 (77.3)	17 (77.3)
Age (years)	36.5 (31.5, 41.8)	35.0 (32.3, 41.8)	39.0 (31.0, 41.0)
BMI (kg/m^2^)	41.4 (37.3, 45.1)^C^	40.7 (36.5, 46.1) ^C^	23.4 (21.7, 24.4)^A,B^
Sleep Apnea, with diagnosis (n (%))	8 (36.4)	7 (31.8)	0 (0) ^A,B^
Scandinavian Descent (n (%))	21 (95.5)	20 (90.9)	22 (100)
Currently Employed (n (%))	4 (18.2) ^B,C^	18 (81.8) ^A^	22 (100.0) ^A^
Waist-to-hip ratio	1.04 (0.95, 1.06)^C^	0.99 (0.92, 1.04)^C^	0.79 (0.76, 0.84) ^A,B^
Fat Percentage (%)	50.2 (47.0, 51.5) ^C^	48.8 (44.8, 51.9) ^C^	23.4 (19.2, 27.7) ^A,B^
Chronotype			
- Definitely Morning (n (%))	2 (9.1%)	1 (4.5%)	3 (13.6%)
- Moderate Morning (n (%))	8 (36.4%)	10 (45.5%)	11 (50.0%)
- Intermediate (n (%))	10 (45.5%)	10 (45.5%)	6 (27.3%)
- Moderate Evening (n (%))	2 (9.1%)	1 (4.5%)	2 (9.1%)

Baseline characteristics of participants with schizophrenia and obesity (SCH-OB), obesity without mental illness (OB), and normal-weight individuals without obesity or mental illness (LEAN). Data are presented as median (IQR) or n (%). Group comparisons were conducted using Kruskal–Wallis tests for continuous variables and unadjusted Mann–Whitney U tests for post hoc pairwise comparisons. Chronotype was estimated using the Morningness-Eveningness Questionnaire. Superscripts indicate significant differences (*p* < 0.05) between groups: ^A^ = different from SCH-OB, ^B^ = different from OB, ^C^ = different from LEAN. Abbreviations: *SCH-OB* Participants with schizophrenia and obesity, *OB* Participants with obesity, *LEAN* Participants with normal weight.

### Clock gene rhythmicity confirmed in 5 out of 6 clock genes, with attenuated PER3 amplitude in schizophrenia

**Fig. 2:**
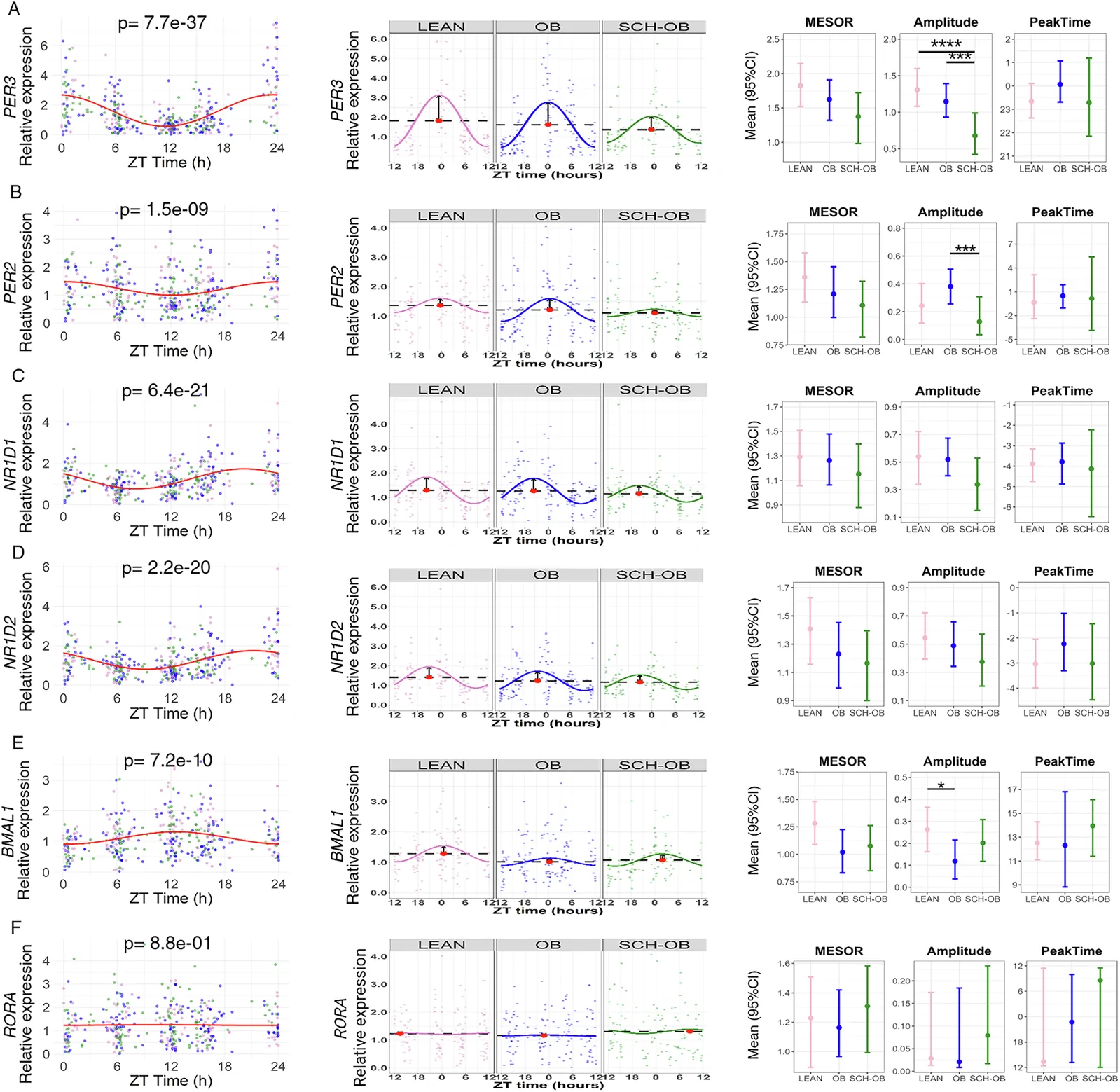
Circadian expression of Clock genes. Panels are arranged vertically by variable. **(A)** *PER3*, **(B)** *PER2*, **(C)** *NR1D1*, **(D)** *NR1D2*, **(E)** *BMAL1*, **(F)** *RORA*. The leftmost panels show pooled model checks of rhythmicity for each gene. P-values reflect likelihood ratio tests comparing rhythmic (harmonic) and non-rhythmic (flat) nested models. Zeitgeber time (ZT time) represents the relative time of day anchored to each participant’s sleep offset (ZT0 = objectively measured individual wake time). P-values are presented in scientific notation to allow comparison across highly significant results; p-values < 0.05 are considered statistically significant. The red curve represents the harmonic regression fit assuming a 24-hour period. The middle panels display group-specific cosinor model fits for each gene. Fitted 24-hour rhythms are shown as solid lines for each group. Dotted horizontal lines indicate the MESOR (Midline Estimating Statistic of Rhythm, i.e., rhythm-adjusted mean); vertical lines represent amplitude (half the peak-to-trough distance); red dots mark the estimated peak time (acrophase, the time of highest expression). These parameters were estimated using linear mixed-effect cosinor models with group included as a fixed effect. The right panels show between-group comparisons of MESOR, amplitude, and peak time based on the same mixed-effects models. Estimated marginal means and 95% confidence intervals were derived via parametric bootstrapping (n = 1000). Asterisks indicate statistically significant differences between groups: * *p* < 0.05, ** *p* < 0.01, *** *p* < 0.001, **** *p* < 0.0001.

We confirmed rhythmic expression of five clock genes: *PER3* (*p* < 0.001; ΔAIC = 162.3), *PER2* (*p* = 1.48 × 10⁻⁹; ΔAIC = 36.6), *NR1D1* (*p* = 6.38 × 10⁻²¹; ΔAIC = 89.0), *NR1D2* (*p* = 2.22 × 10⁻²⁰; ΔAIC = 86.5), and *BMAL1* (*p* = 7.15 × 10⁻¹⁰; ΔAIC = 38.1). Rhythmicity could not be confirmed for *RORA* (*p* = 0.877; ΔAIC = –3.7) (Fig. 2, leftmost panels).

Participants in the SCH-OB group showed lower amplitude of *PER3* compared to both OB and LEAN groups (Fig. 2A, rightmost panel). OB had higher *PER2* amplitude than SCH-OB (Fig. 2B, rightmost panel), while LEAN showed a higher amplitude of *BMAL1* compared to OB (Fig. 2E, rightmost panels). No significant group differences were observed in MESOR or time of peak expression for any of the tested clock genes.

### Schizophrenia is associated with reduced melatonin amplitude and oral pH but intact cortisol rhythms

**Fig. 3:**
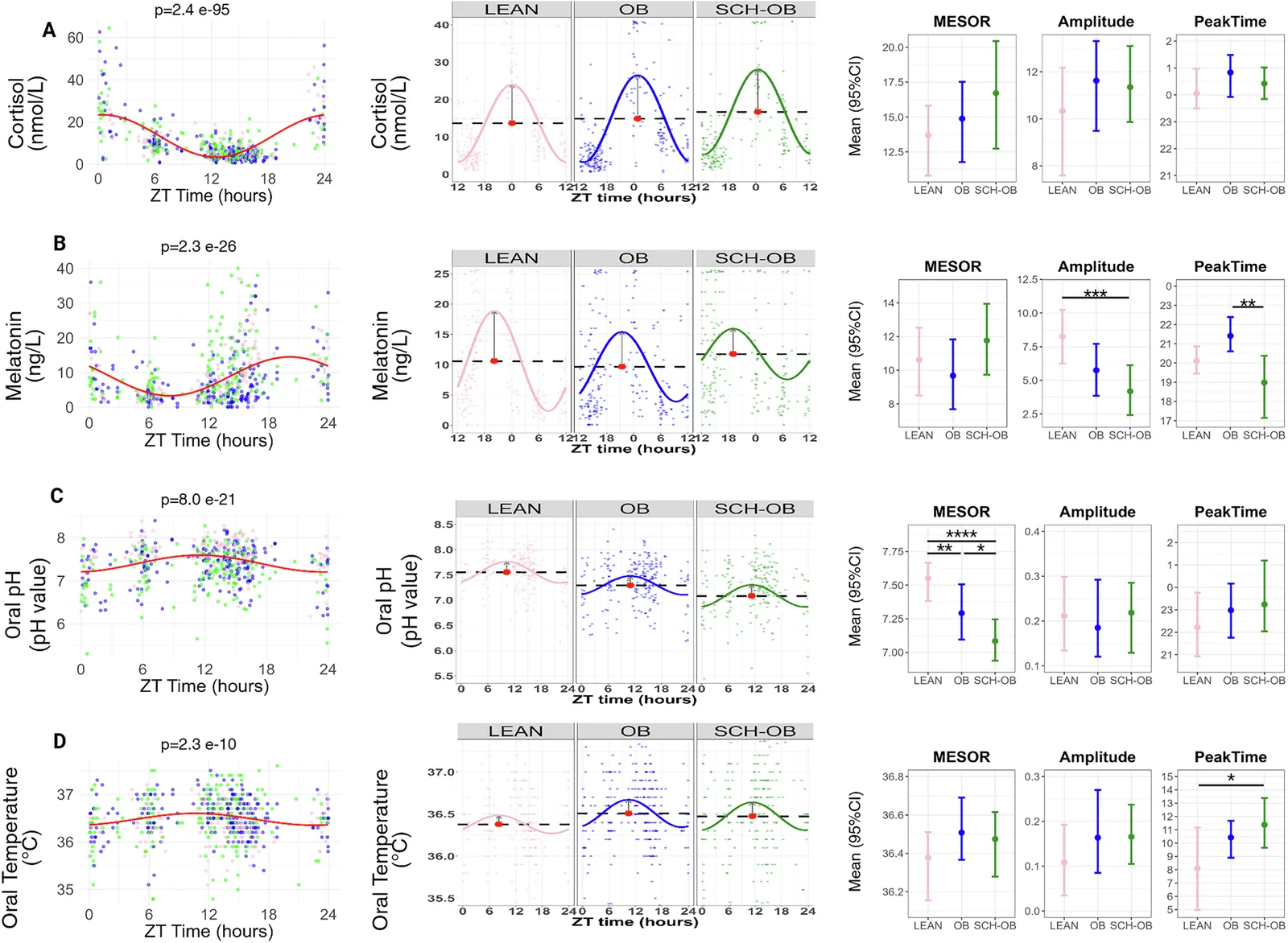
Secondary circadian rhythms in salivary biomarkers and oral physiology. Panels are arranged by variables: **(A)** Salivary cortisol, **(B)** Salivary melatonin, **(C)** Oral pH, and **(D)** Oral temperature. The leftmost plots in each row display rhythmicity testing results. P-values reflect likelihood ratio tests comparing rhythmic (harmonic) and non-rhythmic (flat) nested models. Zeitgeber time (ZT time) represents the relative time of day anchored to each participant’s sleep offset (ZT0 = individual wake time). P-values are presented in scientific notation to facilitate comparison across highly significant results; p-values < 0.05 are considered statistically significant. The red curve represents the harmonic regression fit, assuming a 24-hour period. The middle plots show group-specific fits from linear mixed-effects cosinor models. Fitted 24-hour rhythms are shown as solid lines for each group. Dotted horizontal lines indicate the MESOR (Midline Estimating Statistic of Rhythm, i.e., rhythm-adjusted mean); vertical lines represent amplitude (half the peak-to-trough distance); red dots mark the estimated peak time (acrophase). The rightmost plots display between-group comparisons of MESOR, amplitude, and peak time, presented as estimated marginal means with 95% confidence intervals. Asterisks indicate statistically significant group differences: * *p* < 0.05, ** *p* < 0.01, *** *p* < 0.001.

Both melatonin and cortisol exhibited clear 24-hour rhythmicity. Although sampling was limited to waking hours, model-based fits were broadly consistent with expected biological patterns: melatonin typically peaks during the night [36], while cortisol follows a diurnal rhythm with peak levels shortly after awakening [37]. Consistent with this, cortisol concentrations peaked in the morning across all groups, with no significant group differences in rhythmic parameters (Fig. 3A). Melatonin peak times were estimated to occur approximately 18–22 h after individual sleep offset across all groups, with earlier peaks observed in the OB group compared to the SCH-OB group. Moreover, the amplitude of the melatonin rhythm was significantly reduced in the SCH-OB compared to the LEAN group (Fig. 3B). Additional melatonin and cortisol metrics are provided in the Supplementary Material (Tables S1–2 and Figs. S1–2).

Oral pH and temperature showed 24-hour rhythmicity. pH MESOR was highest in LEAN, intermediate in OB, and lowest in SCH-OB, with all between-group differences significant (Fig. 3C). Oral temperature peaked 8–12 h after sleep offset, earlier in LEAN than SCH-OB, with no significant group differences in amplitude or MESOR (Fig. 3D).

### Objective and subjective sleep measures reveal irregular and poor-quality sleep in schizophrenia

**Fig. 4:**
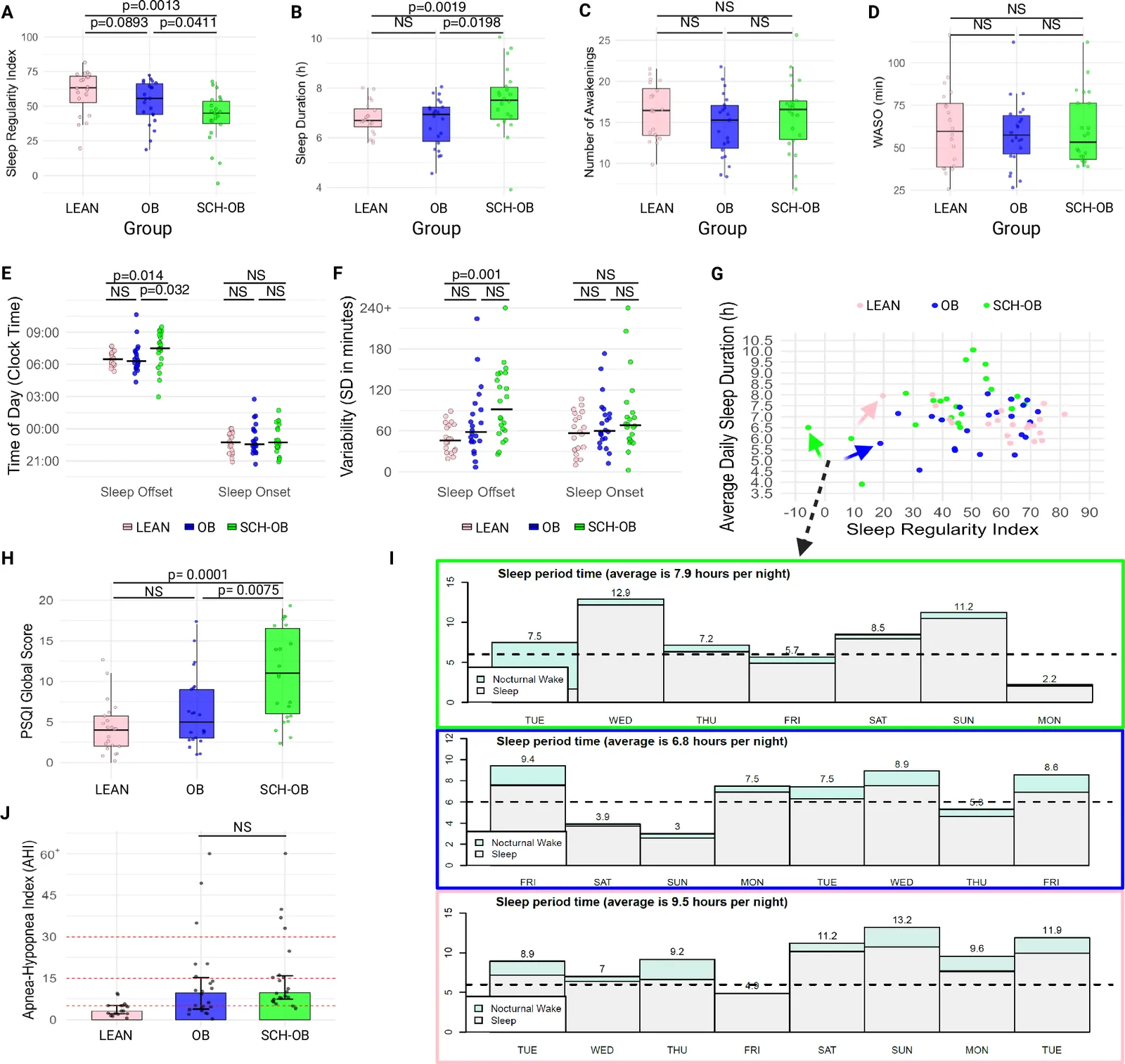
Objective and subjective sleep measures. (**A**–**F**) Accelerometer-derived sleep measures based on mean values across the 8-day sampling period, excluding non-wear time. P-values reflect unadjusted between-group differences assessed using nonparametric Mann–Whitney U tests; NS = not significant. **(A)** Sleep regularity assessed as mean day-to-day regularity over the 8-day period. **(B)** Total sleep duration. **(C)** Number of awakenings after sleep onset. (**D)** Wake after sleep onset (WASO), in minutes. (**E**) Mean wake-up time across the sampling period. **(F)** Variability in individual sleep onset and offset times across the sampling period, expressed as the standard deviation (SD) of timing for each participant. Each point represents an individual’s within-subject variability in sleep onset or offset timing; horizontal lines indicate group-level medians. **(G)** Relationship between SRI and average daily sleep duration; arrows mark the three participants illustrated in (**I**). **(H)** Sleep quality assessed with the Pittsburgh Sleep Quality Index (PSQI). **(I)** Example actigraphy recordings from the participants with the lowest Sleep Regularity Index (SRI) in each group. Top: SCH-OB participant (green arrow in panel G) exhibiting severely irregular sleep, including abrupt shifts in sleep timing and multiple very short sleep episodes. Middle: OB participant (blue arrow in panel G) showing highly variable sleep timing and frequent periods of prolonged nocturnal wakefulness. Bottom: LEAN participant (pink arrow in panel G) with less extreme day-to-day variation in timing, but notable for extended wakefulness during intended sleep periods. **(J)** Sleep apnea screening results based on a single night of cardiorespiratory monitoring. Red dotted lines indicate clinical cutoffs used to define apnea severity categories: mild (AHI ≥ 5), moderate (AHI ≥ 15), and severe (AHI ≥ 30).

SCH-OB participants showed significantly more irregular sleep patterns, reflected by lower SRI values across accelerometry recordings (Fig. 4A), and had longer sleep periods than OB and LEAN (Fig. 4B). No group differences were observed in the number of awakenings or wake after sleep onset (WASO) (Fig. 4C, D). Median sleep onset time was similar across all groups, while sleep offsets appeared later for the SCH-OB group compared to OB and LEAN groups. Sleep offsets were highly variable in the SCH-OB group and a significant contributor to the low SRI in this group (Fig. 4E), though outliers with very short nocturnal sleep periods in the SCH-OB group added further to the low mean SRI in the SCH-OB group (Fig. 4G and I).

Participants in the SCH-OB group reported significantly worse sleep quality than both the OB and LEAN groups, as measured by the Pittsburgh Sleep Quality Index (PSQI).

One SCH-OB participant was diagnosed with OSA post-enrollment, resulting in a minor deviation from the intended OSA matching; however, groups did not differ significantly in OSA severity based on continuous Apnea–Hypopnea Index (AHI) (Fig. 4J).

### Individual-level rhythmicity is most frequently detected in PER3

**Fig. 5:**
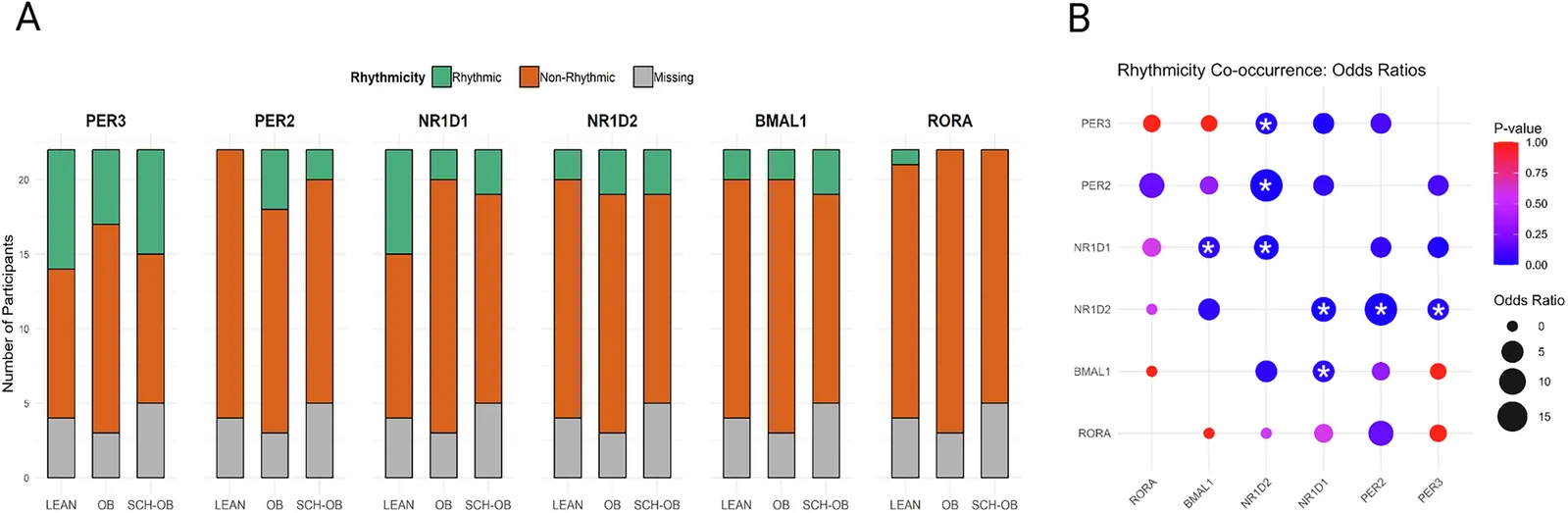
Individual-Level Tests of Rhythmicity in Core Clock Genes. (**A**) Number of participants with rhythmic expression for each core clock gene (*PER3, PER2, NR1D1, NR1D2, BMAL1, and RORA*), determined using individual-level cosinor models. Rhythmicity was assessed using an ANOVA-based F-test comparing a harmonic (cosinor) model to a flat (null) model. Bars are color-coded by rhythmicity status: green = rhythmic (*p* < 0.05), orange = non-rhythmic (p ≥ 0.05), and gray = missing or insufficient data (fewer than 4 valid timepoints). (**B**) Pairwise associations in rhythmic expression between clock genes were tested using Fisher’s exact test. Each bubble represents a gene pair. Bubble size reflects the odds ratio (OR) of Gene A being rhythmic if Gene B is rhythmic; color indicates the raw p-value. Significance was assessed using false discovery rate adjusted p-values, with a threshold of *p* < 0.05.

In order to evaluate the potential clinical utility of hair follicle clock gene analysis, we assessed whether rhythmic parameters could be reliably estimated at the individual level. Participants were included in individual-level cosinor analyses only if at least four viable samples were available. In total, 55 of 66 participants (83%) met this criterion (SCH-OB: n = 17; OB: n = 19; LEAN: n = 19). In the 55 participants with sufficient data for model fitting (≥4 viable samples, Supplementary Fig. S3) and using a conventional significance threshold of *p* < 0.05, we observed rhythmic expression in 20/55 participants for *PER3*, 6/55 participants for *PER2*, 12/55 participants for *NR1D1*, 8/55 participants for *NR1D2*, and 7/55 participants for *BMAL1* (Fig. 5A). In addition, we tested whether rhythmic expression in one gene was associated with rhythmicity in other clock genes, we conducted pairwise analyses across all five rhythmically expressed genes. Interestingly, rhythmic expression in *PER3* was not associated with an increased likelihood of rhythmicity in any gene other than *NR1D1* (Fig. 5B), despite *PER3* being the gene with the highest rate of individual-level rhythmic detection. Individual-level model fits and example profiles are shown in Supplementary Figs S3–S9.

To further contextualize these individual-level detection rates and the accuracy of phase estimation at the individual level, we performed simulation-based phase-precision analyses calibrated to the observed PER3 amplitude and within-person residual variability. Overall, results were consistent with phase precision being primarily driven by the empirical amplitude-to-noise relationship, with only modest gains from extending the number of sampling days at 4 samples/day (Supplementary Figs. S10, S11).

### Higher PER3 amplitude is associated with more regular sleep, but not sleep quality or antipsychotic dose

We investigated whether clock gene amplitude varied as a function of SRI, subjective sleep quality (PSQI), and antipsychotic medication dose, calculated as chlorpromazine equivalent units (CPZeq) [34] (Supplementary Table S3).

A significant positive association was observed between SRI and *PER3* amplitude (p = 0.006), which remained significant after correction for multiple comparisons (adjusted p = 0.030). A nominal association was observed between PSQI and *BMAL1* amplitude (p = 0.032), though this did not survive correction for multiple testing (Supplementary Fig. S12). No associations were found between CPZeq and the amplitude of any clock gene.

## Discussion

This study examined circadian peripheral clock gene expression using hair follicle sampling in individuals with schizophrenia and obesity, individuals with obesity, and healthy controls. Using a repeated sampling protocol under real-world conditions, we found attenuated *PER3* amplitude in schizophrenia compared with matched participants both with and without obesity. We also observed a significant association between *PER3* amplitude and the SRI, linking molecular and behavioral rhythmicity. Hair follicle self-sampling was feasible and yielded sufficient data for individual-level rhythm assessment in the majority of participants. To our knowledge, this is the first study to assess circadian gene expression in individuals with obesity or schizophrenia using repeated sampling outside controlled laboratory settings.

### PER3 amplitude in hair follicles as a marker of circadian rhythms

Clock gene expression profiles derived from hair follicles have mainly been used to determine phase shifts in relation to sleep disorders [38], shift work [19, 39] and chronotype [40, 41]. The *PER3* gene has emerged as the most utilized and reliable marker of circadian phase [42]. Supporting this, Watanabe et al. [43] identified *PER3* as the clock gene exhibiting rhythmicity most consistently across individuals, and notably found no significant *PER3* phase difference when comparing its expression in leukocytes and hair follicle cells. This cross-tissue concordance supports the validity of using hair follicle–derived *PER3* rhythms as a potential marker of systemic circadian timing [43].

While *PER3* is frequently studied for its phase properties, its amplitude, reflecting the strength of its rhythmic expression, has received comparatively little attention. One exception is Takahashi et al., who found that the amounts of physical activity and peak oxygen uptake in older adults were positively correlated with *PER3* amplitude, suggesting that physical activity may help sustain, or even improve, peripheral clock rhythms [41]. In our study, *PER3* amplitude was significantly reduced in individuals with schizophrenia and showed a positive association with the SRI, indicating that irregular rest-activity patterns may contribute to blunted rhythmic output. This interpretation is consistent with findings from Wulff et al. [44], who observed severe circadian and sleep-wake disruption in individuals with schizophrenia, including highly variable sleep timing and desynchronized melatonin rhythms in a substantial subset of individuals. Notably, Wulff et al. restricted their sample to unemployed individuals, indicating that marked irregularity in sleep timing can persist even in the absence of employment-related scheduling constraints. In a supplementary sensitivity analysis, adding employment status as a covariate did not improve model fit (LRT p = 0.54). Taken together, these results point to *PER3* amplitude as a peripheral clock readout of sleep–wake regularity, with particular relevance in psychiatric populations where sleep–wake schedules are often more irregular [44]. In contrast, subjective sleep quality did not show any association with *PER3* amplitude, suggesting that objective measures of sleep–wake regularity may be more closely aligned with molecular rhythm parameters than perceived sleep quality. This may indicate that *PER3* amplitude relates more to sleep regulation and behavioral timing stability than to subjective sleep disturbance, consistent with *PER3* polymorphism studies linking PER3 variation to homeostatic sleep markers without clear differences in melatonin/cortisol rhythms or peripheral PER3 phase [45].

### Distinction between obesity-only and schizophrenia groups

While the OB group showed higher *PER2* amplitude than the SCH-OB group and lower *BMAL1* amplitude than the LEAN group, these isolated differences did not mirror the broader *PER3* amplitude attenuation observed in the SCH-OB group. This distinction suggests that schizophrenia is associated with additional alterations in clock gene rhythm parameters beyond the effects of metabolic burden alone. In line with this, model-based estimates suggested earlier melatonin peak timing in the OB group than in SCH-OB (relative to sleep offset), while actigraphy showed delayed sleep offset in the SCH-OB group compared with both control groups, pointing to a broader desynchronization of behavioral and hormonal rhythms in schizophrenia. In mice, quetiapine or ziprasidone treatment have been shown to increase *PER2* and *BMAL1* expression [46, 47]. When controlling for the use of antipsychotic medication, we found no indication that psychotropic medication use in the SCH-OB group attenuated *PER3* amplitude. This indicates that the blunted rhythmicity could be ascribed to the disease itself or to life-style factors rather than to the use of antipsychotic medication. Supporting this interpretation, Sun et al. (2016) reported persistently reduced *PER3* amplitude in leukocytes of patients with schizophrenia, which remained unchanged following 8 weeks of clozapine treatment despite marked symptomatic improvement [14].

### Salivary hormones and oral rhythms

Salivary melatonin and cortisol rhythms were largely preserved across groups, except for a modest reduction in melatonin amplitude in the SCH-OB group. This suggests that molecular markers of circadian rhythms may be more vulnerable or differently regulated than systemic hormonal outputs. Prior studies have reported highly inconsistent melatonin profiles in schizophrenia, including elevated, reduced, and unchanged levels compared to control individuals [48], highlighting the clinical and biological heterogeneity of the disorder and limiting the utility of melatonin as a uniform circadian marker. Similarly, while salivary cortisol holds value as a noninvasive indicator of hypothalamic-pituitary-adrenal (HPA) axis function [49], elevated levels are more often associated with vulnerability traits and may emerge prior to symptom onset rather than as a stable feature of schizophrenia itself [50]. The absence of inter-group cortisol alterations in the present study supports the view that HPA axis dysregulation, as assessed by salivary cortisol, is not an inherent or persistent feature of schizophrenia [51].

We included oral temperature and pH measures based on prior evidence suggesting that both exhibit 24-hour rhythmic patterns and may reflect underlying physiological processes, [52–54]. In addition to their biological relevance, these measures were chosen for their low cost, minimal participant burden, and ease of integration into field-based investigations. To support adherence to the manufacturer’s recommended passive saliva collection time, we selected a digital thermometer with a measurement duration that naturally occupied the same interval, helping to ensure sufficient saliva for analysis when these were measured concurrently [21].

Lower oral pH has previously been observed in individuals with mental disorders [55]. Our findings align with this but also suggest that obesity may exert a similar contributing effect. The consistently low pH observed across groups with elevated BMI highlights a potential metabolic influence on oral acid–base balance that remains underexplored [56]. This is particularly relevant given that poor oral health is more common among people with mental disorders and is also linked to increased risk of cardiovascular, respiratory, and metabolic diseases — primarily through shared vulnerabilities such as health-detrimental behaviors, chronic inflammation, and systemic immune activation [57]. If oral pH is used as a screening tool for assessing oral health, or for identifying health conditions that influence or are influenced by oral health, our and other studies indicate the importance of adjusting for circadian variation in oral pH [52–54].

### Feasibility of at-home sampling

Adherence to the sampling protocol was high across groups, and the RNA yield was consistently sufficient for qPCR analysis, even in participants with schizophrenia. This shows the method is feasible in vulnerable populations. While overall compliance was strong, participant feedback pointed to sampling-associated discomfort and practical challenges related to repeated hair follicle sampling, particularly over consecutive days. Other studies have utilized hair follicle self-sampling [39, 58–61], but none reported success rate or mean yield, nor compared assisted with self-sampling.

We and others have noted a higher RNA yield in male participants, likely due to stronger follicular adhesion in beard hairs [19, 62]. Bracci et al. found that pubic hair in female nurses yielded the highest RNA quality and quantity among the tested sites, though still requiring 15 follicles per sample [59]. At the same time, thicker, more strongly rooted hairs can increase discomfort, highlighting the need for researchers to balance RNA yield with participant burden when designing sampling protocols.

### Methodological reflections: rhythm detection at individual and group level

While group-level analyses revealed clear rhythmicity in *PER3* expression, rhythm detection at the individual level was more variable. In our samples, one third (20/66) of the participants exhibited statistically significant *PER3* rhythms based on cosinor fitting, with lower detection rates for other genes. Previous studies using similar protocols have produced varying results on individual rhythm detection. For example, Watanabe et al. detected *PER3* rhythmicity in hair follicles of 6 of 8 participants, but rhythmic expression of *PER2* and *BMAL* in only 2/8 and 3/8, respectively [43], using a composite 24/12-hour cosinor model in ChronoLab [63]. Hattammaru et al. reported significant *PER3* rhythms in hair follicles in 64% of daytime workers and 0% of consecutive-night shift workers [39] using the acro.exe [64]. Haraguchi et al. [38] displayed clear rhythms in all participants (with no formal test) but excluded 12/27 participants based on criteria of yield <20 ng/μL or qPCR Ct-values > 33. These findings highlight both biological and methodological factors that constrain rhythm detection at the individual level. Technical limitations, including limited sampling density, variable sample quality, and gene-specific expression amplitudes, can obscure otherwise intact biological rhythms. Our supplementary simulation-based phase-precision analyses indicate that, under the present hair-follicle sampling design, individual-level phase estimation has limited precision and improves only modestly when extending the number of sampling days (Supplementary Fig. S10). One should accordingly be cautious when interpreting failure to detect rhythmicity as evidence of arrhythmicity or altered circadian function. Failure to meet statistical thresholds may reflect low amplitude, irregular timing, or technical noise, rather than true arrhythmicity.

A further challenge lies in the lack of consensus on the optimal method for detecting rhythmicity. A variety of statistical tools are available, each with different assumptions and sensitivities. Beyond classical cosinor analysis, other commonly used methods include spectral approaches such as the Lomb-Scargle periodogram [65] and non-parametric alternatives like the Jonckheere–Terpstra–Kendall test [66]. No gold standard exists, and detection performance can vary substantially depending on sample size, data resolution, waveform shape, and noise characteristics. Consequently, differences in reported rhythmicity across studies may reflect not only biological variation, but also methodological heterogeneity.

### Limitations

Several limitations should be acknowledged when interpreting our findings. First, while our sampling design was optimized for ecological validity, participants collected only four samples per day across two days, which may be insufficient for robust rhythm detection at the individual level, particularly for lower amplitude genes. Second, although the RNA yield was generally adequate, a subset of samples fell below our quantity threshold, and the quantity threshold utilized was lower than those in some other studies [38, 41]. Third, participants in the schizophrenia group were clinically stable and independently living, and findings may not generalize to individuals in acute phases or with more severe functional impairment. Fourth, although antipsychotic medication use was recorded, data on duration of exposure were not available, and our sample size did not permit stratified analysis by medication class or dose, or polypharmacy. Fifth, while we matched participants on key variables like age, sex, BMI, and sleep apnea status, residual confounding from unmeasured lifestyle or pharmacological factors cannot be ruled out. Moreover, the lack of a schizophrenia group without obesity limits inference about obesity-independent effects of schizophrenia. Sixth, contrary to our initial protocol [21], melatonin-based phase estimates were not derived from formal DLMO calculations; therefore, melatonin results should be interpreted cautiously and not as definitive measures of circadian phase. Finally, the cross-sectional design of this study limits causal inference, and future longitudinal or interventional studies are needed to determine whether sleep regularity or other behavioral factors can causally influence circadian gene expression.

### Clinical implications and future directions

Our findings raise the possibility that *PER3* amplitude and sleep regularity may serve as peripheral markers of circadian vulnerability in schizophrenia. The association between the SRI and *PER3* rhythmicity suggests a potential bio-behavioral feedback loop, in which irregular sleep weakens molecular rhythms, while dampened rhythms in turn impair behavioral stability. Given this, interventions that target sleep timing, such as light therapy, behavioral sleep strategies, or structured daily routines, may not only improve subjective and functional outcomes [67], but also strengthen underlying circadian biology. Moreover, aligning the timing of other behavioral cues, such as meal timing and physical activity, with the body’s natural rhythms could further support circadian entrainment and enhance the effectiveness of such interventions [38, 41]. Future longitudinal or interventional studies could incorporate hair follicle sampling to evaluate group-level changes in clock gene expression in response to such treatments, offering a minimally invasive and ecologically valid method for tracking biological rhythms in real-world settings. However, at the present sampling density, hair-follicle sampling appears unlikely to provide clinically reliable individual-level phase (acrophase) estimation.

### Conclusion

Our findings demonstrate that repeated hair follicle self-sampling is a feasible and informative approach for assessing circadian gene expression. We identified a significantly attenuated amplitude of *PER3* rhythmicity in individuals with schizophrenia and found that reduced *PER3* amplitude was associated with lower sleep regularity, linking molecular rhythms to behavioral patterns. These results support the utility of *PER3* amplitude as a potential marker of dampened molecular rhythmicity, while underscoring the need for future studies to refine molecular detection methods and evaluate responsiveness to behavioral or therapeutic interventions.

## Supplementary information

Supplementary Information

## Data Availability

The datasets generated and analyzed in this study are available from the corresponding author on reasonable request. Access to individual-level data requires prior approval from the Danish Data Protection Agency and will be considered case by case.
