# Supplementary material for: Attenuated PER3 amplitude in hair follicles is associated with sleep irregularity in schizophrenia
Source: Transl Psychiatry. 2026 Jul 29;16:584. doi: 10.1038/s41398-026-04295-w (PMC13710538; doi:10.1038/s41398-026-04295-w)
Supplement: Supplementary file 1 [file 41398_2026_4295_MOESM1_ESM.docx]

# Supplementary Information for:

**Attenuated PER3 amplitude in hair follicles is associated with sleep irregularity in schizophrenia**

**Journal:** Submitted to *Molecular Psychiatry*
**ClinicalTrials.gov:** NCT05413486

**Correspondence:** *Mikkel E. I. Kolind,* — Mikkel.Emil.Iwanoff.Kolind@rsyd.dk
Esbjerg Sygehus, Endokrinologisk Afdeling

Haraldsgade 7D, 6700 Esbjerg, Denmark

Content

[Supplementary Information for	1](#_Toc224162227)

[Salivary hormonal concentrations – additional statistics	3](#_Toc224162228)

[Outliers identification	3](#_Toc224162229)

[Cortisol Slope Calculation	4](#_Toc224162230)

[Statistical Results: Cortisol Slope Comparisons	5](#_Toc224162231)

[Psychiatric medication	7](#_Toc224162232)

[Individual-Level Clock Gene Rhythm Analysis	8](#_Toc224162233)

[Simulation-based evaluation of individual-level rhythm detectability and acrophase precision under extended sampling periods	15](#_Toc224162234)

[Associations between Sleep, Medication, and Clock Gene Amplitude	18](#_Toc224162235)

[References	19](#_Toc224162236)

# Salivary hormonal concentrations – additional statistics

We had originally planned to include additional metrics for salivary cortisol and melatonin. Specifically, the wake-to-bed slope for cortisol and the dim-light-melatonin-onset (DLMO) for melatonin ^1^. Saliva samples were collected and processed according to a recently established protocol ^2^ with no noted protocol deviation.

Midday concentrations frequently exceeded 10 ng/L—well above the established DLMO threshold of 3–4 ng/L—making reliable onset detection unfeasible. The 3k method, which requires three consecutive low daytime samples to establish an individualized baseline, could also not be applied ^3^. As extensively discussed by Kennaway, many commercial ELISA kits are prone to overestimating daytime melatonin levels due to limited assay specificity and matrix effects ^4^.

Additionally, both melatonin and cortisol showed frequent outliers. We reviewed these cases individually, consulting sampling times, food diaries, and home-visit notes. Despite these efforts, no plausible sources of contamination were identified. Outliers were therefore handled statistically by excluding extreme values on a timepoint- and group-specific basis to reflect natural group variability. Given limitations in measurement reliability—particularly at the individual level—cortisol statistics are included here for completeness but omitted from the main manuscript.

## Outliers identification

We used a cut point of mean +/- 2 SD, to identify outliers. The mean was taken for all available samples at a given sampling time – as only the first 3 samples were taken at similar times for all participants (0,6,12 hours waking) the later samples were bracketed according to their timing relative to individual bedtimes.

Specifically, samples collected between 0.5 and 1.5 hours before bedtime were labeled as "1h before bed," and the sample collected immediately before going to bed was labeled “bedtime” . This binning allowed samples collected later in the day to be grouped consistently across participants. Outlier detection was then performed separately within each time bin using the mean ± 2 SD criterion. The results of outlier removal is presented in Table 1 and figure 1.

Supplemental Table S1 – Outliers identified within each group and time bin

| **Group** | **Sample Time Bin (approximate ZT_time)** | **Outliers (sample Count)** |  | **Group** | **Sample Time Bin** | **Outlier (sample Count)** |
| --- | --- | --- | --- | --- | --- | --- |
| OB | 0 | 1 |  | OB | 0 | 2 |
| OB | 6 | 2 |  | OB | 6 | 4 |
| OB | 12 | 3 |  | OB | 12 | 3 |
| OB | 1h before bed | 1 |  | OB | 1h before bed | 2 |
| OB | bedtime | 3 |  | OB | bedtime | 2 |
| SCH-OB | 1 | 4 |  | SCH-OB | 0 | 2 |
| SCH-OB | 2 | 2 |  | SCH-OB | 6 | 3 |
| SCH-OB | 3 | 2 |  | SCH-OB | 12 | 2 |
| SCH-OB | 1h before bed | 1 |  | SCH-OB | 1h before bed | 1 |
| SCH-OB | bedtime | 1 |  | SCH-OB | bedtime | 0 |
| LEAN | 0 | 3 |  | LEAN | 0 | 3 |
| LEAN | 6 | 2 |  | LEAN | 6 | 3 |
| LEAN | 12 | 1 |  | LEAN | 12 | 1 |
| LEAN | 1h before bed | 1 |  | LEAN | 1h before bed | 2 |
| LEAN | bedtime | 3 |  | LEAN | bedtime | 1 |

Number of outliers identified within each group and sample time bin based on the mean ± 2 SD method. Outlier detection was performed separately for each group (SCH-OB, OB, LEAN) and for each designated time bin.

**Supplemental Figure S1. Diurnal salivary hormone concentrations**. 24-hour profiles of salivary cortisol and melatonin across two consecutive days, with panels organized by hormone (rows) and data inclusion (columns). (A) Cortisol concentrations including all available data. (B) Cortisol concentrations after outlier exclusion. (C) Melatonin concentrations including all data. (D) Melatonin concentrations after outlier exclusion. Each plot includes five timepoints per day (D1S1 to D1-Bed and D2S1 to D2-Bed), with group means ±1 SD shown for SCH-OB-OB (green), OB (blue), LEAN (pink), and the combined sample (black). Outliers were identified separately within each time bin using a mean ± 2 SD criterion, where the group mean was calculated across all available values at a given timepoint. SCH-OB-OB = SCH-OBizophrenia with obesity; OB = obesity without mental illness; LEAN = lean controls.

## Cortisol Slope Calculation

Cortisol slopes were calculated as the rate of change in cortisol concentration (nmol/L per hour) from the wake sample (first sample of the day) to either 12 hours post-wake (“wake-to-evening slope”) or the final pre-sleep sample (“wake-to-bed slope”). Slopes were computed separately for Day 1 and Day 2 using mean cortisol values within the relevant timepoint bins. If one or both required samples were missing for a given day, the slope was not calculated for that day. Individual slopes were then averaged across days to yield one slope per participant per slope type. Analyses were conducted both with and without excluding hormonal outliers. Table 2 displays group-level slope estimates with and without outlier exclusion

Supplemental Table S2: Cortisol slopes before and after outlier removal:

| **Cortisol wake-to-bed slope** | | | | **Cortisol wake-to-evening slope** | | | |
| --- | --- | --- | --- | --- | --- | --- | --- |
| **Group** | **Mean (With Outliers)**  **nmol/l/hour** | **Mean (No Outliers)**  **nmol/l/hour** | **N Change** | **Group** | **Mean (With Outliers)**  **nmol/l/hour** | **Mean (No Outliers)**  **nmol/l/hour** | **N Change** |
| **OB** | -1.00 ± 0.68 | -0.93 ± 0.48 | 20 → 19 | **OB** | -1.26 ± 0.88 | -1.16 ± 0.59 | 21 → 21 |
| **SCH-OB-OB** | -0.89 ± 0.82 | -0.66 ± 0.38 | 17 → 15 | **SCH-OB-OB** | -1.23 ± 1.07 | -0.92 ± 0.53 | 20 → 18 |
| **LEAN** | -1.03 ± 0.31 | -0.95 ± 0.25 | 21 → 20 | **LEAN** | -1.16 ± 0.55 | -1.09 ± 0.35 | 21 → 20 |

Mean (± SD) cortisol slopes are shown for each group, calculated from wake to evening (12 hours post-wake) and from wake to bedtime. Values are reported separately for analyses with and without hormonal outliers. Slopes represent the rate of change in cortisol (nmol/L per hour). N Change indicates the number of participants contributing valid slopes before and after outlier exclusion. OB = obesity without mental illness; SCH-OB-OB = SCH-OBizophrenia with obesity; LEAN = lean healthy controls.

**Supplemental Figure 2. Cortisol slopes from wake to evening and wake to bedtime, shown by group.** Lines represent mean cortisol change (nmol/L/hour) for each group, plotted from wake (0 h) to either 12 hours post-wake (wake-to-evening slope; left) or to the estimated group-average bedtime (wake-to-bed slope; right). Colors indicate group membership: SCH-OB-OB (green), OB (blue), LEAN (pink). OB = obesity without mental illness; SCH-OB-OB = SCH-OBizophrenia with obesity; LEAN = lean healthy controls.

### Statistical Results: Cortisol Slope Comparisons

A Kruskal–Wallis test was used to assess group differences in cortisol slopes. No significant group differences were found for the wake-to-evening slope, either with outliers included (χ²(2) = 0.765, p = 0.6823) or outliers excluded (χ²(2) = 2.061, p = 0.3568). For the wake-to-bed slope, the test was not significant when outliers were included (χ²(2) = 3.802, p = 0.1494), but became significant when outliers were excluded (χ²(2) = 6.369, p = 0.0414).

Post hoc pairwise comparisons (unadjusted Mann–Whitney U tests) for the wake-to-bed slope (with outliers removed) revealed a significant difference between LEAN and SCH-OB-OB (p = 0.0061), but not between LEAN and OB (p = 0.9668) or OB and SCH-OB-OB (p = 0.0963).

# Psychiatric medication

To enable consistent comparison of antipsychotic exposure across study participants, we calculated chlorpromazine equivalent (CPZeq) doses for all prescribed antipsychotic medications. We used the standardized conversion values reported by Leucht et al., which apply the classical mean dose method based on mean doses from randomized controlled trials ^5^. For participants receiving more than one antipsychotic, CPZ equivalents were summed. Additional medications, including antidepressants, hypnotics, mood stabilizers, muscle relaxants and sleep aids are listed separately for descriptive purposes and were not included in the CPZeq totals (table 1)

**Supplemental Table S3.** Chlorpromazine-equivalent doses (CPZeq) for antipsychotic medications

| Treatment regiment | | | | Additional medications | | | |
| --- | --- | --- | --- | --- | --- | --- | --- |
| CPZeq | Drug (s) | Delivery | Dose, mg | Other psychotropic medications, muscle relaxants and sleep aids | | | |
| 0 | Quetiapine | Oral/daily | 100 |  |  |  |  |
| 116 | Quetiapine Paliperidone | Oral/daily Depot/4-weekly | 100 100 | Lyrica, 75mg, night |  |  |  |
| 125 | Paliperidone | Depot/4-weekly | 150 |  |  |  |  |
| 33 | Aripiprazole | Oral/daily | 5 |  |  |  |  |
| 67 | Aripiprazole | Oral/daily | 10 |  |  |  |  |
| 100 | Aripiprazole | Oral/daily | 15 |  |  |  |  |
| 33 | Clozapine | Oral/daily | 100 |  |  |  |  |
| 0 | - | - | - | Melatonin, 3mg, evening |  |  |  |
| 108 | Clozapine Aripiprazole | Oral/daily | 25 15 |  |  |  |  |
| 0 | - | - | - | Sertralin, 100 mg, evening |  |  |  |
| 0 | - | - | - | Vortioxetin, 10 mg evening |  |  |  |
| 33 | Quetiapine | Oral/daily | 100 | Circadin, 2 mg, evening (PN*) |  |  |  |
| 60 | Risperidone | Oral/daily | 3 | Melatonin, 3mg, evening |  |  |  |
| 67 | Aripiprazole | Oral/daily | 10 | Sumatriptan, 50mg |  |  |  |
| 100 | Aripiprazole | Depot/4-weekly | 400 |  |  |  |  |
| 8 | Quetiapine | Oral/daily | 25 | Sertralin, 150 mg, morning |  |  |  |
| 75 | Xeplion (paliperidon) | Depot/4-weekly | 75 | Ritalin, 10mg, evening |  |  |  |
| 33 | Quetiapine | Oral/daily | 100 | Morphine 10mg (PN*) |  |  |  |
| 100 | Serdolect (Sertindole) | Oral/daily | 4 | Melatonin, 3mg, evening |  |  |  |
| 0 | - | - | - | Sertralin, 200 mg, morning | Lamotrigine, 100 mg, morning | Chlorzoxazon, 500 mg | Naltrexon, 9mg, morning |
| 100 | Abilify Maintena | Depot/4-weekly | 400 |  | Melatonin, 3mg, evening |  |  |
| 41 | Quetiapine Aripiprazole | Oral/daily  Oral/daily | 25  5 |  |  |  |  |

Antipsychotic treatment and chlorpromazine-equivalent doses (CPZeq) for each participant. CPZeq values were calculated using the classical mean dose method from Leucht et al. (2015). For combination therapy, equivalents were summed. Other psychotropic medications are listed descriptively. Abbreviation: CPZeq = chlorpromazine equivalent dose; Depot/4-weekly = long-acting injectable formulation given every 4 weeks; PN = *pro re nata* (as needed).

# Individual-Level Clock Gene Rhythm Analysis

To evaluate the feasibility of detecting circadian rhythmicity at the individual level, we performed separate cosinor analyses for each participant and gene. A minimum of four valid sampling time points was required to support reliable model fitting. This threshold was based on the fact that with only three time points, even random variation can produce an artificially perfect cosine fit, preventing meaningful evaluation of rhythmicity or noise detection. In contrast, four or more samples provide minimal curve constraint and allow some sensitivity to deviations from a sinusoidal shape. Rhythmicity was defined as a significant cosinor model fit based on an F-test comparing the full model (sine and cosine terms) to a MESOR-only model. For the purposes of descriptive classification, we used a liberal threshold of p < 0.10 to sort profiles as “rhythmic” versus “non-rhythmic,” reflecting the limited number of observations available per individual; This threshold was not used for formal inference, but to aid presentation of borderline individual profiles under sparse sampling conditions.

In the illustrative examples shown below, we report the F-test p-value in addition to the R², and RMSE to aid interpretation of fit quality and model performance across varying data conditions.

**Supplemental Figure S3. examples of individual level rhytm detection**. (A) A clear rhythmic expression profile with strong model support (p < 0.001). (B) Four samples collected mainly at time points with limited variability, producing an apparently good fit (visual inspection) but reduced interpretability. (C) A low-amplitude fit near the significance threshold (p = 0.058), highlighting uncertainty in rhythm detection when amplitude is low. (D–E) Illustrations of the limitations of three-sample fits, which yield perfect cosine curves (R² = 1, RMSE = 0) regardless of the underlying signal. (F) An example of non-rhythmic expression; although sampling times were similar across days, values differed markedly, indicating potential biological instability or technical variation, as the majority of available samples are collected outside peak times this further obscures rhythm detection.

**Supplemental Figure S4. Individual-level rhythmicity in Period 3 (PER3) gene expression.** Each panel shows relative PER3 expression plotted across Zeitgeber Time (ZT), overlaid with the individual cosinor fit (blue line). Rhythmicity was assessed for each participant using an F-test comparing the full cosinor model to a null (MESOR-only) model. Participants are grouped into three categories based on test outcome: Top: Rhythmic (p < 0.1); Middle: Not rhythmic (p ≥ 0.1); Bottom: Insufficient data for testing (fewer than four valid time points).

Each **Supplemental Figure S5. Participant-level tests of rhythmic expression of Period 2 gene (PER2)**

panel shows relative PER2 expression plotted across Zeitgeber Time (ZT), overlaid with the individual cosinor fit (blue line). Rhythmicity was assessed for each participant using an F-test comparing the full cosinor model to a null (MESOR-only) model. Participants are grouped into three categories based on test outcome: Top: Rhythmic (p < 0.1); Middle: Not rhythmic (p ≥ 0.1); Bottom: Insufficient data for testing (fewer than four valid time points).

**Supplemental Figure S6. Participant-level tests of rhythmic expression of Nuclear receptor subfamily 1 group D member 1 gene (NR1D1)**

panel shows relative PER2 expression plotted across Zeitgeber Time (ZT), overlaid with the individual cosinor fit (blue line). Rhythmicity was assessed for each participant using an F-test comparing the full cosinor model to a null (MESOR-only) model. Participants are grouped into three categories based on test outcome: Top: Rhythmic (p < 0.1); Middle: Not rhythmic (p ≥ 0.1); Bottom: Insufficient data for testing (fewer than four valid time points).

**Supplemental Figure S7. Participant-level tests of rhythmic expression of Nuclear receptor subfamily 1 group D member 2 gene (NR1D2)**

panel shows relative NR1D2 expression plotted across Zeitgeber Time (ZT), overlaid with the individual cosinor fit (blue line). Rhythmicity was assessed for each participant using an F-test comparing the full cosinor model to a null (MESOR-only) model. Participants are grouped into three categories based on test outcome: Top: Rhythmic (p < 0.1); Middle: Not rhythmic (p ≥ 0.1); Bottom: Insufficient data for testing (fewer than four valid time points).

**Supplemental Figure S8. Participant-level tests of rhythmic expression of Brain and muscle ARNT-like 1 gene (BMAL1)**

panel shows relative BMAL1 expression plotted across Zeitgeber Time (ZT), overlaid with the individual cosinor fit (blue line). Rhythmicity was assessed for each participant using an F-test comparing the full cosinor model to a null (MESOR-only) model. Participants are grouped into three categories based on test outcome: Top: Rhythmic (p < 0.1); Middle: Not rhythmic (p ≥ 0.1); Bottom: Insufficient data for testing (fewer than four valid time points).

**Supplemental Figure S9. Participant-level tests of rhythmic expression of RAR-related orphan receptor A gene (RORA)**

panel shows relative RORA expression plotted across Zeitgeber Time (ZT), overlaid with the individual cosinor fit (blue line). Rhythmicity was assessed for each participant using an F-test comparing the full cosinor model to a null (MESOR-only) model. Participants are grouped into three categories based on test outcome: Top: Rhythmic (p < 0.1); Middle: Not rhythmic (p ≥ 0.1); Bottom: Insufficient data for testing (fewer than four valid time points).

## Factors associated with individual-level PER3 rhythm detection

We examined whether individual-level *PER3* rhythm detection was associated with technical sample characteristics. Participants classified as rhythmic versus non-rhythmic for PER3 expression did not differ in median RNA yield (Wilcoxon p = 0.531) or in the number of viable samples available for model fitting (median 7 viable samples in both groups; p = 0.417).

In contrast, *PER3* rhythm detection was strongly associated with the amplitude-to-noise ratio, defined as individual-level *PER3* amplitude divided by model RMSE. The median amplitude/RMSE ratio was 3.76 in participants classified as rhythmic compared with 1.47 in those classified as non-rhythmic (p ≈ 1.6 × 10⁻⁶). These findings suggest that individual-level rhythm detection was primarily constrained by signal strength relative to residual variability, rather than by RNA quantity alone. Residual variability may reflect a combination of technical variation, and true biological or behavioral variability under home-based sampling conditions.

# Simulation-based evaluation of individual-level rhythm detectability and acrophase precision under extended sampling periods

To quantify (i) the feasibility of detecting individual-level circadian rhythmicity and (ii) the achievable precision of individual peak-time (acrophase) estimates under the present sampling framework—and how both outcomes change with extended sampling duration—we performed two complementary simulation analyses calibrated to the observed PER3 signal characteristics and within-person variability.

**Simulation framework and calibration**

We simulated PER3 expression time series for each group (LEAN, OB, SCH-OB) using a 24-hour cosinor data-generating model with continuous time since first wake (t in hours):

y(t) = MESOR + A·cos(2πt/24 − φ) + ε, ε ~ N(0, σ^2^)

For each simulated individual, amplitude (A), acrophase (phi), and noise level (sigma) were sampled from empirical distributions derived from individual-level PER3 cosinor fits in the observed dataset (Amplitude, Acrophase, and RMSE respectively; individuals required >= 3 valid samples for parameter extraction). Sampling times followed the study protocol pattern (4 samples/day; within-day offsets derived empirically from observed sampling times anchored to wake), repeated for D days (D = 2-6). Where indicated, we additionally introduced missingness by independently dropping each planned sample with probability p (e.g., p = 0.10 corresponds to 10% of planned samples missing), and we recorded the proportion of simulated individuals remaining classifiable for rhythmicity testing (>= 4 retained samples).

**1) Simulation-based detectability of individual rhythmicity with extended sampling**

Aim. To assess how sampling duration (2-6 days) and missing samples influence the probability of detecting individual rhythmicity using the same statistical definition applied in the main analyses.

Model fitting and rhythmicity test. For each simulated individual dataset, we fitted (i) a MESOR-only model and (ii) a 24-hour cosinor model:

- Null model: Y ~ 1
- Cosinor model: Y ~ cos((2*π*t)/24) + sin((2*π*t)/24)

Rhythmicity was evaluated using a nested-model F-test comparing the full cosinor model to the MESOR-only model, and classified as detected when p < 0.05. This mirrors the individual-level rhythmicity test used for observed data (cosinor vs null model). Each scenario (group x days x missingness level) was simulated with n_sim = 1000 individuals.

Outcome metrics. For each group and sampling duration, we report (1) the proportion of simulated individuals remaining classifiable (>= 4 retained samples) and (2) the proportion with rhythmicity detected (p < 0.05).

Interpretation. Under complete sampling, extending collection from 2 to 6 days increased the probability of detecting rhythmicity at the individual level, consistent with improved model constraint as the number of observations increases. Introducing missing samples reduced both classifiability and rhythm detection probability—particularly at shorter sampling durations—highlighting that incomplete sampling can substantially limit individual-level rhythm classification even when the underlying rhythm is present. Overall, these results support that increasing the number of sampling days can improve detectability, but the magnitude of improvement depends strongly on data completeness.

**Supplementary figure S10: Rhythm detectability; faceted by missingness).**

Simulation-derived probability of detecting individual PER3 rhythmicity (nested-model F-test, p < 0.05; cosinor vs MESOR-only) under sampling schedules repeated across 2-6 days, shown separately for LEAN, OB, and SCH-OB groups. Facets indicate the proportion of planned samples missing in the simulation (e.g., 'Missing: 20%' corresponds to each planned sample being dropped with probability 0.20). Lines show the proportion of simulated individuals meeting the rhythmicity criterion; results also account for classifiability (>= 4 retained samples).

**2) Simulation-based acrophase precision with extended sampling duration**

Aim: To quantify what level of individual peak-time (acrophase) precision is achievable under the present sampling protocol (4 timepoints/day x 2-day hair-follicle sampling) and how precision changes with extended sampling duration.

Model fitting and uncertainty estimation. For each simulated individual dataset, we fitted a cosinor regression model:

*Y ~ cos(2*π*t/24) + sin(2*π*t/24)*

Acrophase was computed from the fitted sine/cosine coefficients. Uncertainty was quantified using residual bootstrap with B = 500 resamples per individual. For each individual, we derived a circular 95% confidence interval for acrophase using the shortest arc covering 95% of bootstrap acrophase draws, and summarized precision as the CI half-width (hours). For each scenario (group x days), we simulated n_sim = 1000 individuals.

Outcome metrics. For each group and sampling duration (days), we report the proportion of simulated individuals achieving an acrophase 95% CI half-width <= 1 h, <= 2 h, and <= 4 h.

Interpretation. Across groups, extending the sampling duration from 2 to 6 days increased the proportion of individuals meeting a given acrophase-precision criterion, but gains were modest at a sampling frequency of 4 timepoints/day. The OB group showed consistently lower phase precision than LEAN and SCH-OB under the same sampling schedule, consistent with a lower empirical amplitude-to-noise ratio in the calibrated individual-level fits. Minor non-monotonic variation across day settings is expected due to finite simulation and bootstrap replication (Monte Carlo error). Overall, these results indicate that—under an ecologically valid design with signal-to-noise characteristics similar to the present study—simply extending the number of sampling days is unlikely to yield substantial improvements in individual-level phase determination.

**Supplementary figure S11 – simulated acrophase precision**

Simulation-derived proportions of individuals achieving predefined acrophase precision thresholds under a 4 samples/day hair-follicle sampling schedule repeated across 2–6 days, shown separately for LEAN, OB, and SCH-OB groups. Each panel reports the proportion of simulated individuals whose acrophase 95% CI half-width falls below the specified threshold: ≤1 h (panel A), ≤2 h (panel B), and ≤4 h (panel C).

# Associations between Sleep, Medication, and Clock Gene Amplitude

We evaluated whether clock gene amplitude was associated with subjective sleep quality (Pittsburgh Sleep Quality Index, PSQI), sleep regularity (accelerometer-derived Sleep Regularity Index, SRI), or antipsychotic medication dose, expressed as chlorpromazine equivalents (CPZeq). Supplementary Figure S11 summarizes these associations.

**Supplemental Figure S12. Estimated circadian gene expression amplitude as a function of sleep quality, sleep regularity and psychotropic medication.**

Each panel shows the predicted amplitude of one core clock gene (*PER3*, *PER2*, *NR1D1*, *NR1D2*, *BMAL1*) modeled as a function of Pittsburgh Sleep Quality Index (Column A), Sleep Regularity Index (Column B), or chlorpromazine equivalent dose (Column C). Column C includes only participants with schizophrenia. Mixed-effects cosinor models were used to test whether each predictor modulates circadian amplitude, by including interaction terms between the predictor and the sine and cosine components of circadian time. Participant ID was included as a random intercept to account for repeated measurements. Prediction lines reflect fixed-effect estimates, and shaded ribbons indicate bootstrapped 95% confidence intervals (1,000 simulations). P-values were derived from bootstrapped slopes of the amplitude–predictor relationship and adjusted for multiple comparisons using the Benjamini–Hochberg procedure.

## Exploratory medication-related case review

As a further exploratory medication-related sensitivity check, we reviewed individual SCH-OB cases to evaluate whether low PER3 amplitude appeared to cluster with higher antipsychotic exposure, polypharmacy, or longer time since diagnosis. No clear pattern was observed. Participants with lower PER3 amplitude did not consistently show higher chlorpromazine-equivalent doses, greater medication burden, or longer time since diagnosis. These descriptive case-level observations do not exclude medication effects, but they did not indicate that the observed PER3 amplitude findings were clearly driven by antipsychotic exposure or illness duration.

# References

1.	Kolind ME, Kruse R, Petersen AS, Larsen CS, Bak LK, Højlund K *et al.* Investigating the role of obesity, circadian disturbances and lifestyle factors in people with schizophrenia and bipolar disorder: Study protocol for the SOMBER trial. *Plos one* 2024; **19**(7)**:** e0306408.

2.	Anton J, Larsen CS, Gudmundsson M, Bak LK. The effects of storage conditions on the stability of salivary melatonin in synthetic fiber swabs for home sampling. *Scandinavian Journal of Clinical and Laboratory Investigation* 2025**:** 1-4.

3.	Molina TA, and Burgess HJ. Calculating the Dim Light Melatonin Onset: The Impact of Threshold and Sampling Rate. *Chronobiology International* 2011; **28**(8)**:** 714-718.

4.	Kennaway DJ. A critical review of melatonin assays: Past and present. *J Pineal Res* 2019; **67**(1)**:** e12572.

5.	Leucht S, Samara M, Heres S, Davis JM. Dose Equivalents for Antipsychotic Drugs: The DDD Method. *Schizophr Bull* 2016; **42 Suppl 1**(Suppl 1)**:** S90-94.
